# Scaling Up Family Planning to Reduce Maternal and Child Mortality: The Potential Costs and Benefits of Modern Contraceptive Use in South Africa

**DOI:** 10.1371/journal.pone.0130077

**Published:** 2015-06-15

**Authors:** Lumbwe Chola, Shelley McGee, Aviva Tugendhaft, Eckhart Buchmann, Karen Hofman

**Affiliations:** 1 PRICELESS–MRC/Wits Rural Public Health and Health Transitions Research Unit (Agincourt), School of Public Health, Faculty of Health Sciences, University of the Witwatersrand, Johannesburg, South Africa; 2 Department of Obstetrics and Gynaecology, Faculty of Health Sciences, University of the Witwatersrand, Johannesburg, South Africa; University of Bremen, GERMANY

## Abstract

**Introduction:**

Family planning contributes significantly to the prevention of maternal and child mortality. However, many women still do not use modern contraception and the numbers of unintended pregnancies, abortions and subsequent deaths are high. In this paper, we estimate the service delivery costs of scaling up modern contraception, and the potential impact on maternal, newborn and child survival in South Africa.

**Methods:**

The Family Planning model in Spectrum was used to project the impact of modern contraception on pregnancies, abortions and births in South Africa (2015-2030). The contraceptive prevalence rate (CPR) was increased annually by 0.68 percentage points. The Lives Saved Tool was used to estimate maternal and child deaths, with coverage of essential maternal and child health interventions increasing by 5% annually. A scenario analysis was done to test impacts when: the change in CPR was 0.1% annually; and intervention coverage increased linearly to 99% in 2030.

**Results:**

If CPR increased by 0.68% annually, the number of pregnancies would reduce from 1.3 million in 2014 to one million in 2030. Unintended pregnancies, abortions and births decrease by approximately 20%. Family planning can avert approximately 7,000 newborn and child and 600 maternal deaths. The total annual costs of providing modern contraception in 2030 are estimated to be US$33 million and the cost per user of modern contraception is US$7 per year. The incremental cost per life year gained is US$40 for children and US$1,000 for mothers.

**Conclusion:**

Maternal and child mortality remain high in South Africa, and scaling up family planning together with optimal maternal, newborn and child care is crucial. A huge impact can be made on maternal and child mortality, with a minimal investment per user of modern contraception.

## Introduction

Every year, nearly 3,000 mothers and 40,000 children under five years die in South Africa mainly from preventable causes [1–3]. Although substantial progress has been made in reducing maternal and child mortality in the last few years, this will not be sufficient to reach the millennium development goals (MDGs) 4 and 5 [4]. There is now, more than ever, an urgent need to scale up high impact interventions to save the lives of mothers, newborns and children [5–7]. With just a few months left to the millennium development goals (MDG) deadline in 2015, the focus of the international community is shifting to the post-2015 development agenda, with calls for family planning to be at the core of the post-2015 goals because of its potential to contribute to sustainable development [8, 9]. Family planning offers good value for investment because it is cross-cutting and impacts nearly all the MDGs, including reduction of poverty and hunger, increasing universal education, promotion of gender equality, reduction in maternal and child mortality, reduction in HIV/AIDS and environmental sustainability [10].

The contribution of family planning to maternal and child health cannot be overemphasised. Globally, birth spacing through increased use of modern family planning methods can save the lives of more than 2 million newborns and children every year [11]. Scaling up family planning could prevent one third of maternal deaths by allowing women to delay motherhood, avoid unintended pregnancies and subsequent abortions [12]. In South Africa, there are over 80,000 registered abortions annually, some of which can be potentially avoided with increased family planning [13]. In addition, teenage pregnancy is high with more than 20% of girls between 15 and 19 reporting ‘ever having been pregnant’ [14, 15]. These pregnancies can be avoided by improved family planning. Further, because HIV is the underlying cause in over 40% of maternal deaths, family planning could have a significant impact on maternal mortality. Mother-to-child transmission of HIV can also be reduced, leading to a decline in child mortality.

Despite the benefits, many women in South Africa still do not use modern contraceptive methods. Their use among women 15–49 increased modestly from 62% in 1998 to 64% in 2003, and in that year the unmet need for family planning was measured at 13% [15]. It is thus critical to ramp up efforts to provide universal access to modern contraception, especially if family planning is to be at the core of the post-2015 agenda.

The South African government has demonstrated commitment to expanding its family planning programme. It is party to Family Planning 2020, a global partnership between governments, civil society, donors and other stakeholders, aiming to expand contraceptive use to 120 million more women and girls by 2020 [16]. In line with this commitment, in 2012, South Africa developed a new family planning policy, with emphasis on dual protection (using condoms together with other contraception) [17]. The policy revision sought to update family planning provision to include newer contraceptive methods and in early 2014, new sub-dermal contraception implants were introduced, adding to the available options.

Effective implementation of South Africa’s new family planning programme requires information on the necessary resources needed to expand modern contraceptive use. Such data is, however scant. In 2012, it was estimated that the cost of contraceptive care in the developing world was US$4 billion, and scaling up family planning to meet the need for modern contraception would cost an additional US$8 billion annually [18]. In South Africa, it is estimated that the total cost of family planning for all HIV positive women in 2009 was approximately US$3.3 million [19]. However, a complete picture is required to show the costs and benefits of expanding family planning to all women who need it.

This paper shows the potential costs and benefits of scaling up modern contraception in South Africa. We model the effects of increasing the contraceptive prevalence rate (CPR) on population size, growth rate, pregnancies, births, abortions and maternal and child mortality. This data can inform the implementation of South Africa’s new family planning policy.

## Methods

We used the Spectrum program to model the impact of family planning on maternal and child mortality. Spectrum consists of a suite of modules that are used to assess the impact of interventions on population health. The modules in Spectrum include the Lives Saved Tool (LiST), AIDS Impact Module (AIM), Family Planning (FamPlan) and Demographic Projections (DemProj) [20]. DemProj is the main Spectrum model, which projects country specific populations by age and sex. The other modules interact with DemProj, to address various issues including fertility, HIV/AIDS and maternal and child survival. In this analysis, we use FamPlan and LiST to examine the impact of family planning on fertility rates, pregnancies, births, abortions and maternal and child survival.

FamPlan helps to inform policy on family planning by projecting the requirements needed to address unmet need for modern contraception in a country. The model can be used to set family planning goals, estimate the service expansion needed to meet the goals, and evaluate alternative methods to achieve the goals. FamPlan uses the proximate determinants model of fertility to relate contraception to the total fertility rate. The proximate determinants model of fertility explains the fertility inhibiting factors including contraception, abortion, marriage, postpartum insusceptibility and primary infertility [21, 22]. The model postulates that fertility would be at its highest if these inhibiting factors were non-existent or at a minimum. FamPlan projects the population fertility by considering the effect of each proximate determinant. Thus, inputs of each proximate determinant of fertility are required in the model: the percent of women aged 15–49 years in a sexual union (in our analysis, we consider sexually active women, given that many births in South Africa occur outside informal and formal unions), the proportion using modern contraception by method, the degree of primary infertility, the use of abortion, and the duration of postpartum insusceptibility to conception. Assumptions include the proximate determinants of fertility and the characteristics of the family planning program (method mix, source mix, discontinuation rates) to calculate the cost and the number of users and acceptors of different contraceptive methods by source. FamPlan calculates indicators showing the number of family planning users, commodities required, unplanned pregnancies and births, and numbers of abortions—all based on the data entered into the model, the assumptions made, and the desired outcomes.

In this analysis, the impact of family planning is estimated by increasing the contraceptive prevalence rate (CPR). The CPR, the percentage of sexually active women of reproductive age (15–49 years) using contraceptive methods [15], is used because it is generally easy to measure and a straight forward indicator of the number of contraceptive users. We used a CPR for South Africa of 64.6% at baseline (2014). The CPR was then increased by 0.68% per year in a 15 year period from 2015 to 2030. This was made on the basis of a previously observed change in CPR of approximately 0.68% per year between 1998 and 2003 [15].

### FamPlan inputs and assumptions

#### Proximate determinants

The proximate determinants of fertility for South Africa included in this analysis are obtained mainly from the 2003 Demographic and Health Survey [15] and 2013 World Fertility Report [23], and include: a) proportion of women in sexual union aged 15–49 years (46%); b) postpartum insusceptibility—the period after a birth during which a woman is not exposed to the risk of pregnancy, was 13 months [15]; c) contraception—the level of contraception use by method is given in Table 1. The use of the sub-dermal implant was negligible before 2014, when the National Department of Health began scaling it up. We assumed the use of the implant would increase to 0.5% by the end of 2014, 1% in 2015 and 2016, and to 2% thereafter (with the remaining methods of contraception maintaining the same proportions relative to each other); d) the level of unintended pregnancy terminated or induced abortion was 19%, based on Southern African regional estimates [24]; and e) the prevalence of sterility was 5% [15].

**Table 1:** Percentage use of contraception by method.

Methods	Percent
Male condom	12
Female sterilization	16
Male sterilization	1
Injectable contraceptive	51
Implant (3 years)	0
IUD	1
Oral contraceptive	19

Source: South Africa Demographic and Health Survey, 2003; World Fertility Report, 2013; Spectrum defaults

#### Other assumptions

The unmet need for family planning used was 13% [15], this reflects the difference between women’s contraceptive behaviour and their reproductive intentions. Based on Spectrum, the total fertility rate, the number of children that would be born to a woman if she were to live to the end of her childbearing years, was estimated to be 2.43 in 2014.

#### Contraception costs

Costs of scaling up family planning were modelled in FamPlan, using cost data from the United Nations Population Fund (UNFPA). Details of the costing methodology are provided elsewhere [18]. Costing took an ingredients approach and was undertaken from a health service provider perspective. Both direct and indirect costs are included. Direct costs were estimated for contraceptive commodities, supplies and labour needed for counselling, method provision and supply, follow-up and method removal (where needed). Unit costs are obtained from the Management for Sciences Health International Drug Price Indicator, United Nations Children’s Fund Supply Catalogue and the WHO-CHOICE database. Indirect costs included programme management, supervision, personnel training, health education, monitoring and evaluation, advocacy, systems strengthening and maintenance and expansion of physical capacity for health facilities. All prices were adjusted to 2012 US dollars. The annual unit costs of each contraceptive method are provided in Table 2.

**Table 2:** Unit costs (2012 US dollars) of contraceptive methods per year.

Method	Cost (US$)
Male condom	3.9
Female sterilization	2.79
Male sterilization	1.59
Injectable contraceptive	9.14
Implanon (3 years)	7.74
Oral contraceptive	8.72
IUD	1.01

Source: UNFPA

### Estimating the impact of Family Planning on maternal and child survival

After making adjustments to the FamPlan model in Spectrum, the impact of scaling up family planning on maternal, newborn and child survival can be assessed in LiST [25], which has been extensively used to model the impact of intervention scale up on maternal and child mortality [5, 6, 26]. National baseline information on mortality rates and causes of death, background variables (e.g. fertility, economic status), current coverage of more than 60 interventions and their associated effectiveness values relative to specific causes of death and risk factors are used to estimate the deaths averted, overall and by specific interventions.

**Fig 1:**
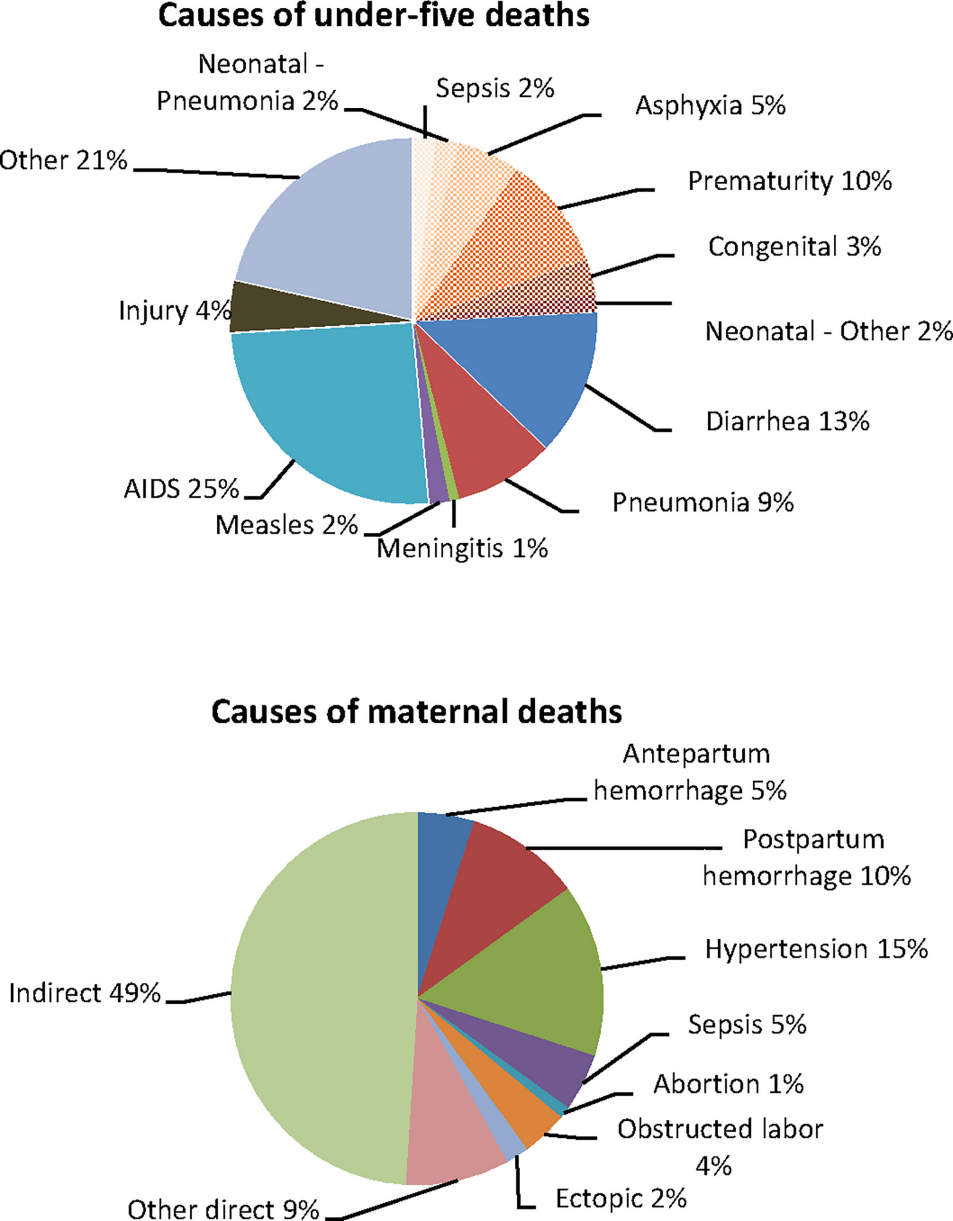
Causes of child, new born and maternal deaths in SA used in the LiST model.

We used a maternal mortality ratio of 269 deaths per 100,000 live births [4], under-five mortality rate of 41/1,000 and neonatal mortality rate of 13/1,000 [3]. The causes of maternal [27], newborn and child [28] mortality used are given in Fig 1.

The baseline coverage of interventions included in LiST was reviewed and where possible compiled using national population based data (Table 3). LiST does not provide default coverage values for all interventions included in the model. Some of these interventions include ectopic pregnancy, management of abortions, pre-eclampsia and clean birth practices. Data on the coverage of these interventions in South Africa are also not available. The coverage levels used in our analysis are based on consensus obtained from local experts across maternal and child health on reasonable coverage levels obtaining in South Africa, taking into consideration recent policy changes, financial and resource inputs, and observed localised coverage change. The consultation with the experts took place at a one day meeting to discuss the trends in maternal and child interventions in South Africa. We invited 23 participants who work in the health sector in various positions at national and district level, clinical practice and academia. The participants deliberated on the coverage levels of each intervention, drawing on their own experiences.

**Table 3:** Baseline (2014) intervention coverage of interventions included in the LiST model.

Interventions	Baseline coverage
Safe abortion services	35
Post abortion case management	60
Ectopic pregnancy case management	40
Antenatal care (4 visits)	50
Tetanus toxoid vaccination	77
Calcium supplementation	5
Hypertensive disease case management	40
Diabetes case management	10
MgSO4—Management of pre-eclampsia	75
Fetal growth restriction detection and management	10
Skilled birth attendance (SBA)	93
Facility delivery (clinic and hospital)	87
Unassisted deliveries	5
Basic emergency obstetric care (BEMOC)	5
Comprehensive emergency obstetric care (CEMOC)	50
Clean birth practices	70
Immediate assessment and stimulation	70
Labour and delivery management	93
Neonatal resuscitation	40
Antenatal corticosteroids for preterm labour	20
Antibiotics for rapture of membrane	25
MgSO4 management of eclampsia	80
Active management of the third stage of labour	80
Induction of labour for pregnancies lasting 41+ weeks	10
Promotion of breastfeeding	25
Preventive postnatal care	10
Clean postnatal practices	10
Complementary feeding—education only	10
Complementary feeding—supplementation and education	5
Vitamin A supplementation	50
Improved water source	91
Water connection in the home	69
Improved sanitation—Utilization of latrines or toilets	74
Hand washing with soap	17
Hygienic disposal of children's stools	41
BCG	74
Polio	74
DPT	66
Hib	66
HepB	74
Pneumococcal	64
Rotavirus	66
Measles	74
Maternal Sepsis case management	75
Kangaroo mother care	25
Case management of severe neonatal infection	44
Injectable antibiotics	70
Full supportive care	44
ORS—oral rehydration solution	50
Antibiotics—for treatment of dysentery	80
Zinc—for treatment of diarrhoea	10
Oral antibiotics: case management of pneumonia in children	73
Vitamin A—for treatment of measles	75
Therapeutic feeding—for severe wasting	45
Treatment for moderate acute malnutrition	10
PMTCT	90
Early treatment of HIV in pregnant women	40
Treatment of TB in pregnant women	50
Treatment of injuries in children 1–5 years	50
Treatment of TB in children 1–5 years	50

In the base case analysis, maternal, newborn and child deaths were then estimated, holding this baseline coverage constant, and compared to a scenario where coverage of all interventions was increased linearly by 0.5% per year. Taking 2014 as the base year, intervention scale up was started in 2015.

The impact of family planning on maternal and child mortality was measured in terms of deaths averted. First, we calculated the expected number of deaths at the current (baseline) level of intervention coverage. Second, the numbers of deaths were recalculated with increased coverage for all interventions in the year 2030 (5% annually). Deaths averted (or additional lives saved) were then estimated by subtracting the numbers of deaths at baseline from the deaths at scale. The deaths averted attributed to family planning were measured by subtracting the number of deaths with and without changes in the level of family planning. We also estimated the potential life years gained, which were calculated as deaths averted multiplied by life expectancy, using a life expectancy at birth of 60 years for newborns and children [29], and a reproductive-aged life expectancy of 27 years for mothers [30].

### Scenario analysis

A scenario analysis was undertaken to test the impact of changes in the base case assumptions, where CPR was increased minimally by 0.1%. Due to data unavailability, this choice was made to represent a lower uptake of family planning. In the scenario analysis, all essential maternal, newborn and child interventions were linearly scaled up until coverage reached 99% in 2030.

## Results

### Base case analysis

The results of this analysis show the impact of increasing the contraceptive prevalence rate (CPR) on the total fertility rate (TFR), births, abortions and maternal and child deaths. Also presented are the total annual costs of scaling up family planning methods by 0.68% per year. The baseline CPR was 64.6% (Table 4), which was projected to increase to 75.5% in 2030 (with CPR increasing by 0.68% per year).

**Table 4:** Base case results for projected demographic events and impact of family planning on maternal, newborn and child mortality.

Projected demographic events	2014 baseline	Changes in 2030
Contraceptive prevalence rate (%)	64.6	75.5
Total fertility rate (number)	2.43	1.65
Total number of pregnancies	1 336 800	1 006 000
Unintended pregnancies (number)	535 400	383 500
Abortions (number)	103 400	74 071
Live births (number)	1 059 600	939 500
Projected impact on mortality	2014 baseline	Changes in 2030
Number of maternal deaths	2 800	1 700
Number of child deaths (0–69)	38 100	28 300
Number of neonatal deaths	12 800	10 800
Maternal mortality ratio (deaths per 100,000 live births)	269	210
Maternal mortality rate (deaths per 10,000 women aged 15–49)	21	11
Under-5 mortality rate (deaths per 1,000 live births)	41	34
Neonatal mortality rate (deaths per 1,000 live births)	12	12
Deaths averted by family planning (2030)	Deaths averted	Potential life years gained
Maternal deaths	600	16 200
Child deaths (0–69 months)	5 900	354 000
Neonatal deaths	1 500	90 000

All essential maternal and child health interventions linearly scaled up by 0.5% per year. Potential life years gained = total deaths multiplied by life expectancy (27 years for mothers and 60 years for neonates and children). CPR = Contraceptive prevalence rate. Figures rounded to the nearest 100.

#### Impact on demographic events and maternal, newborn and child mortality

The TFR was estimated to be 2.43 in 2014, and by 2030, would decline to 1.65; the total number of pregnancies reduce to one million (Table 4), and unintended pregnancies, abortions and births reduce by approximately 23%.

At baseline, before scale up of family planning or other interventions, the total annual number of maternal deaths was estimated to be 2,800 (Table 4). In 2030, this would reduce to approximately 1700 (with scale up of maternal and child interventions by 0.5% per year).

The annual numbers of child deaths reduce from approximately 38,000 in 2014 to 28,000; and the deaths of newborns reduce from 12,800 to 10,800 in 2030.

The maternal mortality ratio (estimated at 269 in 2014) would reduce to 210 maternal deaths per 100,000 live births. The maternal mortality rate (deaths per 10,000 women 15–49 years) reduces from 21/10,000 in 2014, to 12/10,000.

Increasing family planning by 0.68 percentage points per year averted an additional 600 maternal deaths in 2030. The number of child and neonatal deaths averted by family planning were 5,900 and 1,500, respectively (Table 4). The potential life years gained were 16,200 for mothers, 354,000 for children and 90,000 for neonates.

#### Commodity requirements and costs of family planning

Table 5 shows the annual number of units of each contraceptive method required to meet the need when family planning is scaled up. If family planning increases by 0.68 percentage points per year, the contraceptive requirements between 2014 and 2030 increase as follows: required units of male condoms increases from 54 million to 72 million; injectable contraception increases from 7 million to 10 million; the implant increases from 19,200 to 41,800; the oral contraceptive increases from 10 million to 12 million; and IUD from 23,500 to 27,800.

**Table 5:** Base case results for total commodity (number of units) requirements for each contraceptive method per year.

Commodity	2014 baseline	Changes in 2030
Male condom	54 636 800	72 260 200
Injectable contraceptive	7 740 200	10 238 000
Implant	19 270	41 800
Oral contraceptive	10 813 500	12 044 700
IUD	23 500	27 800

CPR = Contraceptive prevalence rate. IUD = Intrauterine device. Figures rounded to the nearest 100.

The total annual costs of providing contraception in 2014 (before scale up) are estimated to be approximately US$26 million. In 2030, the annual costs would be US$33 million (if CPR increases by 0.68% per year).

The annual number of users of modern methods of contraception is projected to be 4 million in 2014, and by 2030 will rise to 5.5 million. The average cost per user per year of modern contraception is about US$7 and the annual cost per capita (total cost/total South African population) is US$0.6. The annual cost of family planning per potential life year gained is US$ 2,000 for mothers, US$79 for children and US$320 for newborns. The incremental cost (2030 costs minus 2014 costs) per death averted is US$2400 for child and US$30,000 for maternal deaths. The incremental cost per life year gained is US$40 for children and US$1,000 for mothers.

### Scenario analysis

Table 6 shows the results of the scenario analysis for the projected demographic events and impact of family planning on maternal, newborn and child mortality (in 2030). The projected TFR is 2.3, the total number of pregnancies are 1.3 million, and maternal deaths are 1,100. The maternal mortality rate (21/10,000 at baseline) is expected to be 8/10,000. Increasing family planning in the scenario analysis would avert 300 maternal, 4,500 child and 1,300 neonatal deaths in 2030 (Table 7).

**Table 6:** Results of scenario analysis for projected demographic events and impact of family planning on maternal, newborn and child mortality (shown are changes in 2030).

Projected demographic events	CPR increases by 0.1%
Contraceptive prevalence rate (%)	66.2
Total fertility rate (number)	2.30
Total number of pregnancies	1 341 100
Unintended pregnancies (number)	533 200
Abortions (number)	103 000
Live births (number)	1 064 000
Projected impact on mortality	CPR increases by 0.1%
Number of maternal deaths	1 100
Number of child deaths (0–69)	18 100
Number of neonatal deaths	4 700
Maternal mortality ratio (deaths per 100,000 live births)	108
Maternal mortality rate (deaths per 100,000 women aged 15–49)	8
Under-5 mortality rate (deaths per 1,000 live births)	17
Neonatal mortality rate (deaths per 1,000 live births)	4

All essential maternal and child health interventions are scaled up to 99% coverage. Potential life years gained = total deaths multiplied by life expectancy (27 years for mothers and 60 years for neonates and children). CPR = Contraceptive prevalence rate. *Results only for CPR increase by 5%. Figures rounded to the nearest 100.

**Table 7:** Results of the scenario analysis for projected deaths averted and potential life years gained by family planning.

Deaths averted	CPR increases by 0.1%	
	Deaths averted	Potential life years gained
Maternal deaths	300	8 100
Child deaths (0–69 months)	4 500	270 000
Neonatal deaths	1 300	78 000

All essential maternal and child health interventions are scaled up to 99% coverage. Potential life years gained = total deaths multiplied by life expectancy (27 years for mothers and 60 years for neonates and children). CPR = Contraceptive prevalence rate. Figures rounded to the nearest 100.

**Fig 2:**
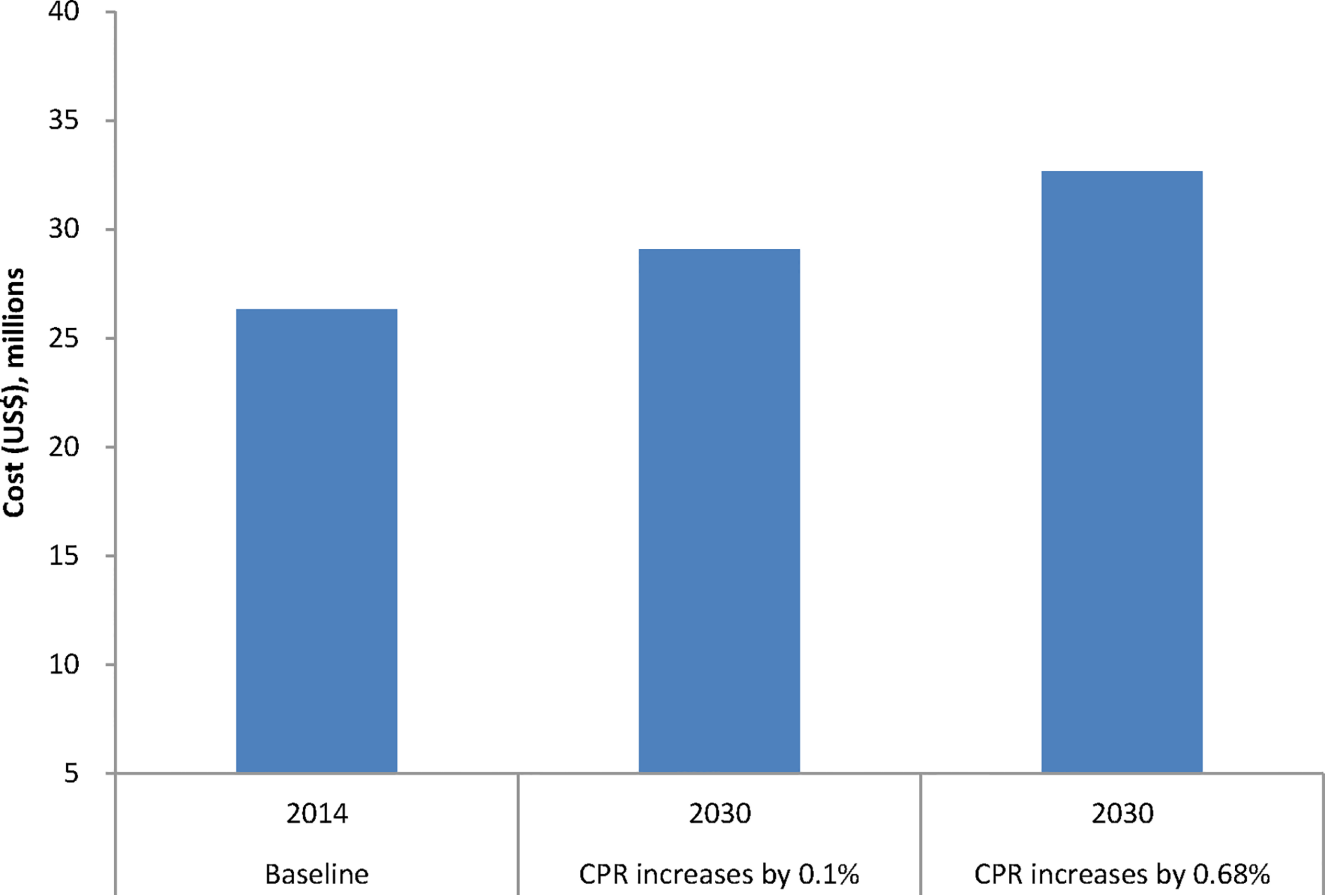
Total annual costs (2012 US$) of family planning projected by the model.

Fig 2 compares the total annual costs of providing contraception in all scenarios. Compared to an estimated cost of US$26 million in 2014, the annual costs of providing contraception would be US$29 million in the scenario analysis (Fig 2).

## Discussion

This paper demonstrates the potential impact of increasing the use of modern contraception on fertility, and maternal, newborn and child survival in South Africa. Using a model for family planning, we scaled up the contraceptive prevalence rate, over a period of 15 years (2015 to 2030), by 0.68 percentage points annually. In a scenario analysis, we tested the impact of increasing CPR by 0.1% annually. In addition, we assumed that maternal, newborn and child interventions to reduce mortality (including emergency obstetric care, antenatal care, kangaroo mother care and breastfeeding promotion) were scaled up by 0.5% annually (and 99% by 2030 in the scenario analysis). The results show that in 2030, unintended pregnancies, abortions and births could reduce by approximately 23% if CPR increases by 0.68% per year. This could avert 7,000 child and newborn deaths and avert 600 maternal deaths per year. Scaling up family planning will have a huge impact on the total fertility rate, which would fall to 1.65. This is far below the population replacement rate, and would make South Africa comparable to countries such as Brazil (1.8) and Singapore (1.3) [31]. The fertility rate at this level would see an eventual decline in population growth in South Africa.

The results on maternal and child survival are made on the basis of aspirational goals (0.5% per year and 99% intervention coverage by 2030), which may not be easy for South Africa to attain in the short-term, and does not take into account the quality of these interventions. However, the rates of scale up were selected in order to show what might be possible if concerted efforts were made to optimise intervention coverage. The rapid increase in coverage of interventions such as skilled attendance at birth, immunisations and PMTCT in the last decade, indicate that attainment of full intervention coverage may be feasible. Similarly, achieving the suggested contraceptive prevalence rates may seem daunting, but it is possible as shown by countries such as Brazil and Thailand, where contraceptive prevalence is above 80% [31].

In this analysis, we have provided an estimate of the commodity requirements and costs should the contraceptive prevalence rate increase. The total annual costs of providing modern contraception are estimated to be US$33 million in the base case analysis and US$29 million (US$0.5 per capita) in the scenario analysis. The cost of scaling up family planning would amount to less than 0.5% of the 2014 national health budget, and about 1% of the current primary healthcare expenditure per capita of US$74 [32]. We, however, cannot state on the basis of this evidence that scaling up family planning is affordable to the South African government. In our results, we provide estimates of cost-effectiveness ratios measured as costs per potential life years gained. These show that scaling up family planning is highly cost-effective, when judged against the gross domestic product per capita threshold [33]. Such information could be used in future analyses to compare the cost-effectiveness of family planning to other maternal and child health interventions.

The results provided in this paper should be taken with caution, as they do not imply that expanding family planning will necessarily lead to an increased use in contraception. There are many barriers to contraceptive use from both the demand and supply side [34, 35]. In South Africa, the leading reasons for not using contraceptives include concerns regarding side effects and opposition by partners [36, 37]. Among adolescents, parental consent is often an issue, and in a country such as South Africa where ‘traditional’ values are entrenched, and sex is in many cases still a taboo topic, sexually active adolescents may not easily access contraception even if it were made freely available [38]. On the supply side, efforts should be made to address inadequate logistics and protocols [39] and strengthen the training of front-line health workers in the provision of family planning [36]. Contraceptives should also be made readily available and the demand for the variety of methods should be met, as this is essential to meeting women’s desire to space births. It is also important to deal with the problem of health worker biases and judgmental attitudes, particularly with regard to adolescents who wish to access family planning services [37].

Addressing these issues could require substantially more resources than the current model is capable of generating, and could significantly lead to higher societal costs. More needs to be done to understand these costs. Furthermore, the costs estimated in this paper are dependent on the contraceptive method mix, and a change in this distribution could impact the associated costs. However, should this be the case in future, the unit costs provided here [18] could be used to make the necessary adjustments. In addition, the costs of scaling up key interventions to reduce maternal, newborn and child mortality have not been included in this analysis, but they should be included in future, since funding for family planning cannot be considered in isolation, but as part of a package of essential interventions.

The full benefits of family planning on the health system could not be entirely addressed in this analysis, yet the consequences may be greater than the impact on mortality. We estimated approximately 1.3 million pregnancies and deliveries in South Africa, which can be reduced by ramping up family planning. With fewer pregnancies and births, more resources could be freed up, potentially leading to an improvement in service delivery for antenatal care and childbirth.

Further, in order to fully realise the benefits of family planning, choices must be made on the appropriate indicators to measure progress and impact. The choice of maternal mortality ratio, instead of maternal mortality rate as the key MDG 5 indicator does not adequately portray the positive role of family planning. As shown in our sensitivity analysis results, the impact of family planning on the maternal mortality ratio remained static even after scaling up family planning in both scenarios. This may be because the absolute number of maternal lives saved is not incorporated in the maternal mortality ratio, since increasing contraceptive prevalence reduces the number of births, the denominator in the ratio. Perhaps a more informative measure would be the maternal mortality rate, whose denominator is the number of women in the reproductive age group.

One of the impediments to effective investment in family planning is insufficient data on the use of modern contraception. Available data on contraceptive prevalence and the unmet need for family planning in South Africa are either outdated or inadequate. The last measurement of national level unmet need was made in the 2003 South Africa Demographic and Health Survey. Several developments have occurred since then, and the need to update these statistics cannot be overemphasized. National data on the method mix of contraceptive use is available in some surveys, and the District Health Information System (DHIS). On the face of it, the DHIS could provide a proxy for contraceptive use, but its reliability is of concern, since it is only collected in health facilities, and contains many inconsistencies. Furthermore, most of the data available, particularly institutional data, are not age-disaggregated or sub-group stratified. Therefore, in this analysis, we used a broad age-group of 15–49 years. A more insightful analysis could have been performed if data in five year age-groups had been available. It is important to take into account the age-distribution of users, as this has an impact on the usage of contraception and the benefits that can be realised from family planning. Another limitation of the Spectrum model is that it does not take into account the impact of birth spacing on child mortality, hence it underestimates the benefits of family planning. The importance of birth spacing to child survival is well documented and studies have shown that higher birth intervals are associated with lower risks of child mortality [11, 40]. Spectrum does not include this relationship, since intervention impact is estimated through the reduction of causes of child death. These are considerations that should be made in future analyses when changes to the model are made to account for such effects.

As we approach the post-2015 era, emphasis should be placed on renewed efforts to reduce maternal and child mortality. The expansion of family planning services in many low and middle income countries, including South Africa has been hampered by shifts in international health and development priorities, and the focus of attention on HIV/AIDS, infectious diseases and poverty alleviation [41]. This has resulted in a disproportionate allocation of resources to vertical programmes such as HIV/AIDS. Greater emphasis should be placed on more comprehensive packages.

## Conclusions

Scaling up family planning can help avert nearly 7,000 newborn and child deaths and 300 maternal deaths annually; with investments of approximately US$7 per user per year. This is probably a minimal estimate because other requirements such as logistics and infrastructure, which impact on costs, have not been considered. As we approach the end line of the millennium development goals, there is great need for an appraisal of the impact of the family planning policy in the last decade, and to find solutions to the many challenges facing its adequate implementation. Significant strides could be made in the post-2015 agenda by focusing on increasing essential maternal and child interventions, and bringing family planning to the fore. We estimate that with concerted efforts and appropriate investment, South Africa can reach its MDG 4 target of 20 child deaths per 1,000 live births by 2030; and also get closer to meeting its MDG 5 target of reducing maternal mortality.
